# Effects of Acid Treatment on Dental Zirconia: An *In Vitro* Study

**DOI:** 10.1371/journal.pone.0136263

**Published:** 2015-08-24

**Authors:** Haifeng Xie, Shuping Shen, Mengke Qian, Feimin Zhang, Chen Chen, Franklin R. Tay

**Affiliations:** 1 Jiangsu Key Laboratory of Oral Diseases, Nanjing Medical University, Department of Prosthodontics, Affiliated Hospital of Stomatology, Nanjing Medical University, Nanjing, China; 2 Jiangsu Key Laboratory of Oral Diseases, Nanjing Medical University, Department of Endodontics, Affiliated Hospital of Stomatology, Nanjing Medical University, Nanjing, China; 3 Department of Endodontics, College of Dental Medicine, Georgia Regents University, Augusta, Georgia, United States of America; The University of Tokyo, JAPAN

## Abstract

The aim of this study was to evaluate the effects of hydrofluoric (HF) acid, acetic acid, and citric acid treatments on the physical properties and structure of yttria-stabilized tetragonal zirconia polycrystal (Y-TZP) at ambient temperature. In total, 110 bar-shaped zirconia specimens were randomly assigned to 11 groups. The specimens in the control group (C) received no surface treatment, while those in the Cage group were hydrothermally aged at 134°C and 0.2 MPa for 20 h. Ten specimens each were immersed at ambient temperature in 5% and 40% HF acid for 2 h (40HF0), 1 day (5HF1, 40HF1), and 5 days (5HF5, 40HF5), while 10 each were immersed at ambient temperature in 10% acetic acid and 20% citric acid for 7 (AC7, CI7) and 14 days (AC14, CI14). X-ray diffraction (XRD) was used to quantitatively estimate the monoclinic phase. Furthermore, flexural strength, surface roughness, and surface Vickers hardness were measured after treatment. Scanning electron microscopy (SEM) was used to characterize the surface morphology. The Cage group specimens exhibited an increased monoclinic phase and flexural strength. Furthermore, 40% HF acid immersion decreased the flexural strength and surface hardness and deteriorated the surface finish, while 5% HF acid immersion only decreased the surface hardness. All the HF acid-immersed specimens showed an etched surface texture on SEM observations, while the other groups did not. These findings suggest that the treatment of Y-TZP with 40% HF acid at ambient temperature causes potential damage, while treatment with 5% HF acid, acetic acid, and citric acid is safe.

## Introduction

Yttria-stabilized tetragonal zirconia polycrystal (Y-TZP) is a popular nonmetallic material used for dental restorations and implants because of its superior mechanical properties and biocompatibility. Prostheses fabricated from Y-TZP are supposed to be more clinically durable than those prepared from other all-ceramic systems. However, clinical studies reported Y-TZP framework fractures in extended fixed dental prostheses and crowns [1,2]. Susceptibility to low-temperature degradation [LTD, based on tetragonal phase (*t*) to monoclinic (*m*) phase transformation] has allegedly been an important factor affecting the mechanical properties and survival of Y-TZP frameworks [3–6]. However, chemical degradation was also recently reported to alter the mechanical properties of Y-TZP in an adverse manner. According to Egilmez et al, the flexural strength of zirconia decreased significantly after immersion in 4% acetic acid at 80°C for 7 days [7]. In other studies, Y-TZP readily corroded after immersion in 100°C HCl/Fe_2_Cl_3_, hydrofluoric acid (HF), nitric acid, and sulfuric acid for 30 min [8,9]. These findings collectively suggest the potential adverse effects of acid solutions on the crystalline structure and mechanical properties of Y-TZP. However, the experiments in these previous studies involved acid etching at high temperatures, not ambient temperature. The oral cavity is a potentially hostile environment because of the high humidity and varied pH values. In particular, an acidic environment caused by the ingestion of beverages and foods with low pH values is predominant, and restorations fabricated from Y-TZP inevitably come into contact with this environment. Furthermore, some authors advocate etching or cleaning of Y-TZP restorations with HF acid before bonding. In a study by Sriamporn et al [10], 9.5% and 48% HF was used to etch dental zirconia ceramic at 25°C, resulting in micromorphological changes. However, that study did not investigate the effects of HF acid etching on the mechanical properties and crystalline structure of Y-TZP. Investigation of the effects of an acidic environment created by the ingestion of low-pH beverages and foods or by HF etching on the physical properties and crystalline structure of Y-TZP at ambient temperature is essential to determine whether such actions should be avoided. Unfortunately, few studies have evaluated these effects. Therefore, the present study was designed to evaluate the effects of HF and two typical acids, citric acid and acetic acid, which come from juice and vinegar, respectively, on the physical properties and phase composition of Y-TZP at ambient temperature and to determine whether these changes occur because of LTD and chemical degradation or chemical degradation only. The null hypothesis tested was that there are no differences in Y-TZP immersed in HF acid, acetic acid, and citric acid for different time periods with respect to *t→m* transformation, destabilization of the crystalline phase, and deterioration of physical properties.

## Materials and Methods

### Specimen preparation

In total, 110 bar-shaped specimens (2 × 5 × 25 mm^3^) were sectioned from a machinable Y-TZP block (zirconia ≤ 94%, alumina ≤ 0.5%; Everest ZS-B42/16, KAVO, Altenbach & Voigt GmbH, Bismarcking, Germany) using an electric low-speed saw (Isomet 100, Buehler Ltd., Lake Bluff, IL, USA). The specimens were wet polished with 600- and 800-grit silicon carbide abrasive papers and completely sintered in a crystallization furnace (EverestTherm, KAVO) at a temperature of 1450°C for 2 h according to the manufacturer’s instructions. The dimensions of the zirconia specimens after sintering were approximately 1.2 × 4 × 20 mm^3^.

The Y-TZP bars were randomly assigned to 11 groups (N = 10). Specimens in the control group (C) received no further surface treatment, while those in the Cage group were hydrothermally aged at 134°C and 0.2 MPa in an autoclave (Vacuklav 24B, MELAG, Germany) for 20 h. Ten specimens each were immersed at ambient temperature in 5% and 40% HF for 2 h (40HF0), 1 day (5HF1, 40HF1), and 5 days (5HF5, 40HF5). The other specimens (N = 10) were immersed in 10% acetic acid and 20% citric acid for 7 (AC7, CI7) and 14 days (AC14, CI14). The treatments of all the experimental groups were summarized in Table 1. After treatment, all specimens were ultrasonically cleaned in distilled water for 10 min before testing.

**Table 1:** Summarization of the treatments for all the experimental groups.

Groups	Treatments
**C**	Control, no treatment
**Cage**	Control, hydrothermally aged for 20 hours
**40HF0**	40% HF immersed for 2 hours
**40HF1**	40% HF immersed for 1 day
**40HF5**	40% HF immersed for 5 days
**5HF1**	5% HF immersed for 1 day
**5HF5**	5% HF immersed for 5 days
**AC7**	Acetic acid immersed for 7 days
**CI7**	Citric acid immersed for 7 days
**AC14**	Acetic acid immersed for 14 days
**CI14**	Citric acid immersed for 14 days

### Flexural strength, surface roughness, and surface Vickers hardness evaluation

The 110 Y-TZP bars were subjected to three-point bending tests performed using a universal testing machine (Instron Model 3365, Norwood, MA, USA) equipped with a three-point bending jig that had a span length of 14 mm. Prior to testing, the dimensions of each specimen were determined using a digital micrometer. Moreover, all edges were rounded off along the long axis of the specimen and provided a 0.1-mm-wide chamfer, as proposed by ISO 6872 [11]. Each specimen was loaded mid-length with a crosshead speed of 1 mm/min until failure, and the load at fracture was recorded. The flexural strength (in MPa) was calculated using the equation M = 3FL/2WT^2^, where F is the load at fracture, L is the span length (center-to-center distance between the supporting rollers), W is the specimen width, and T is the specimen thickness.

The reliability of flexural strength testing was assessed using the Weibull distribution [12–14]. For each of the 11 test groups, the stress values were ranked in ascending order as follows: i = 1, 2, 3, …, N), where N is the total number of test specimens and i is the ith datum. Accordingly, the lowest stress value for each configuration is represented by the first datum (i = 1), the second lowest value is represented by the second datum (i = 2), and so on, with the highest stress value represented by the Nth datum. This enables the determination of a ranked probability of failure, P_f_ (σ_f_), which is assigned to each datum according to the following formula:

$$P_{f}=\;\left(i\;-\;0.5\right)/N$$

Then, least-squares estimation (LSE), maximum likelihood estimation (MLE), and mean and variance evaluation were used to evaluate each material and treatment group using the scale parameter σ_θ_ and the Weibull modulus m (two-parameter Weibull distribution) according to the following formula:

$$P_{f}=\;1\;-\;exp\{-{\left(\sigma_{f}/\sigma_{\theta }\right)}^{m}\},$$

where P_f_ is the probability of failure at or below the stress σ_f_.

The following calculations were derived from the above equation:

$$lnln[1/(1-P_{f})]\;=\;mln\sigma_{f}-mln\sigma_{\theta }\;\text{l}$$

The plotting of *lnln*[1/(1 − *P*_*f*_)] against *σ*_*f*_ provided a slope with the value m (Weibull modulus) and an intercept m *lnσ*_*θ*_.

Five fractured Y-TZP bars (flexural strength closer to the mean) were selected from each group for the evaluation of surface roughness (Ra) using a surface profilometer (Mahr Perthometer PGK, Mahr GM, Germany). Each surface roughness value was derived from the mean of three different observation points.

The five selected bars were then subjected to surface Vickers hardness testing. Indentations were created using a Vickers microhardness tester (FM-700, Future-Tech Corp., Kanagawa, Japan) with a constant load of 9.807 N for 10 s. The diamond indentations were assessed using scanning electron microscopy (SEM, LEO 1530VP, Oberkochen, Germany) at 15 kV with 5000× magnification.

All the above data are expressed as means, standard deviations, and 95% credibility intervals. Following validation of the normality and homoscedasticity assumptions of the data sets, one-way analysis of variance (ANOVA) and least significant different (LSD) tests (for multiple comparisons) were performed to determine statistically significant differences among groups using the SPSS 19.0 statistical software package (SPSS Inc., Chicago, IL, USA). A p-value of 0.05 was considered statistically significant.

### Morphological analysis

The surface of a selected fractured Y-TZP bar (flexural strength closest to the mean) from each group was sputter-coated with gold for examination using SEM at 20 kV in the secondary electron imaging mode, with magnifications of 30×, 3000×, and 10000×.

### Characterization of phase transformation

The amount of *m*-ZrO_2_ crystals in a random Y-TZP bar selected from each group was determined using X-ray diffraction (XRD; Shimadzu XRD-6000 X-ray diffractometer, Bruker, Germany) with Ni-filtered Cu K radiation (λ = 1.5418 Å) at ambient temperature. The test parameters were as follows: step size, 0.01° 2θ; start angle, 25°; end angle, 80°; and scan speed, 2° 2θ/min. The monoclinic phase weight fraction (*X*_m_) was calculated using the method of Garvie and Nicholson [15].

### Characterization of the sediments obtained after acid immersion

The sediments formed after acid immersion were separated, washed with absolute ethanol, and dried. XRD with the same test parameters mentioned above was used to characterize the dried powders and determine the composition of the mixture. The sediments were assessed by SEM with energy-dispersive X-ray microanalysis (SEM/EDS; 15 kV, 200×; INCAx-sight, Oxford Instruments, United Kingdom). On the basis of the EDS analysis, the oxide weight percent was calculated using stoichiometry.

## Results

### Flexural strength, surface roughness, and surface Vickers hardness values

The flexural strength, surface roughness, and surface Vickers hardness values for each of the 11 groups are shown in Table 2. Two specimens in the 40HF5 and 40HF1 groups, respectively, disintegrated before testing, and their flexural strength values were considered as null strength. The majority of specimens fractured into two parts during flexural strength testing, with only a few breaking into three parts.

**Table 2:** Flexural strength, Vickers hardness, and surface roughness values for each group.

Groups	Flexural strength (MPa)		Surface Vickers hardness		Surface roughness (μm)	
	Mean ± SD	Confidence ıntervals (95%)	Mean ± SD	Confidence ıntervals (95%)	Mean ± SD	Confidence ıntervals (95%)
**C**	1232.04±228.98^a^	1056.03–1408.05	1334.88±11.71^h^	1320.32–1349.43	0.23±0.05^m^	0.12–0.35
**Cage**	1417.55±173.90^b^	1293.15–1541.96	1352.72±21.81^h^	1325.63–1379.80	0.22±0.06^m^	0.06–0.38
**40HF0**	888.90±124.76^c^	799.65–978.15	1128.60±103.02^I^	1000.67–1256.52	0.44±0.19^n^	0.02–0.91
**40HF1**	693.72±273.13^d^	498.33–889.10	1083.00±118.63^I^	935.69–1230.30	0.59±0.03^n^	0.52–0.66
**40HF5**	540.59±242.42^d^	367.16–714.01	1084.80±93.55^I^	968.64–1200.95	1.15±0.14°	0.81–1.50
**5HF1**	1071.03±182.80^a^	948.22–1193.84	1219.60±97.93^J^	1097.99–1341.20	0.35±0.06^m^	0.19–0.51
**5HF5**	1164.29±192.00^a^	1026.94–1301.64	1231.40±55.08^J^	1163.00–1299.79	0.26±0.02^m^	0.20–0.31
**AC7**	1281.57±176.49^ab^	1134.02–1429.13	1337.80±14.28^h^	1320.05–1355.54	0.27±0.06^m^	0.12–0.41
**AC14**	1122.07±273.30^a^	911.98–1332.15	1365.00±49.46^h^	1303.58–1426.41	0.30±0.06^m^	0.17–0.44
**CI7**	1214.44±114.98^a^	1132.19–1296.70	1330.60±9.65^h^	1318.60–1342.59	0.34±0.17^m^	0.07–0.75
**CI14**	1348.50±173.89^ab^	1214.84–1482.17	1397.80±47.11^h^	1339.29–1456.30	0.37±0.09^m^	0.15–0.59

Values with the same superscript letters are not significantly different (*p <* 0.05).

One-way ANOVA revealed significant differences among the 11 groups in flexural strength, surface roughness, and surface Vickers hardness (all p < 0.05) values. LSD tests revealed the following results. The flexural strength decreased in all the HF acid-treated groups except the groups 5HF1 and 5HF5, with the maximum decrease observed in the 40HF5 and 40HF1 groups. The surface roughness increased only in the HF acid-treated groups, with the increase being directly proportional to the concentrations and immersion times. The Vickers hardness decreased only in the HF acid-treated groups, with the decrease being directly proportional to the concentrations.

**Fig 1:**
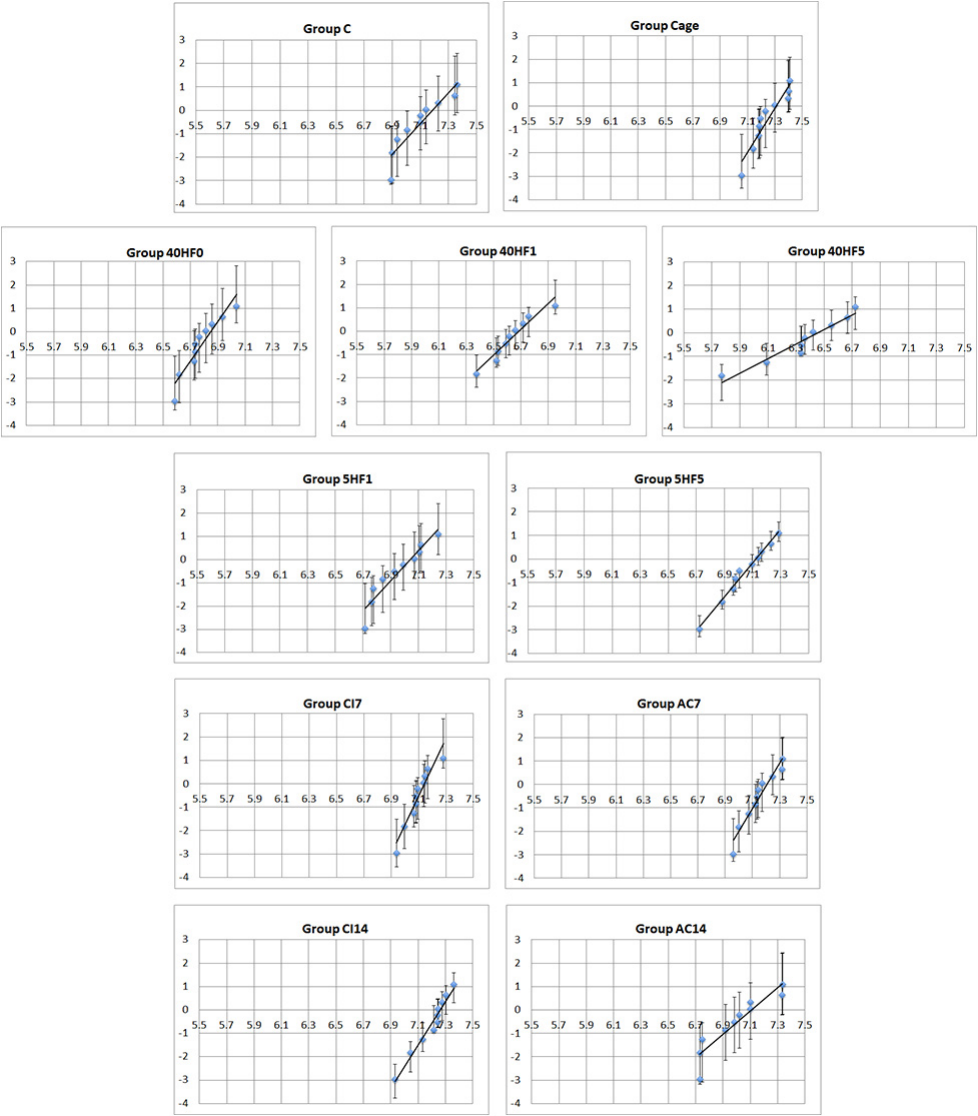
Weibull plots for all the experimental groups with 95% confidence interval.

The Weibull statistical parameters are presented in Table 3. A high Weibull modulus indicated a smaller error range, a higher level of structural integrity, and potentially greater structural reliability of the material. Statistical analysis revealed significant differences among the flexural strength values obtained for all tested groups. The 40HF0, 40HF1, and 40HF5 groups showed the lowest mean flexural strength values and Weibull moduli. The Weibull distribution presented the highest shape values for the CI7, CI14, and Cage groups. A Weibull plots for all the experimental groups with 95% confidence interval was shown in Fig 1.

**Table 3:** Weibull statistics determining a 10% probability of fracture (P10) derived from the flexural strength values.

Groups	^+^P_10_ (MPa)	Scale parameter, σ_θ_ (MPa)	*m
**C**	921	1327	6.2
**Cage**	1176	1493	9.4
**40HF0**	720	941	8.4
**40HF1**	532	801	5.5
**40HF5**	305	636	3.1
**5HF1**	825	1146	6.9
**5HF5**	905	1242	7.1
**AC7**	1038	1356	8.4
**CI7**	1056	1264	12.5
**AC14**	759	1230	4.7
**CI14**	1101	1424	8.7

### Morphological analysis

**Fig 2:**
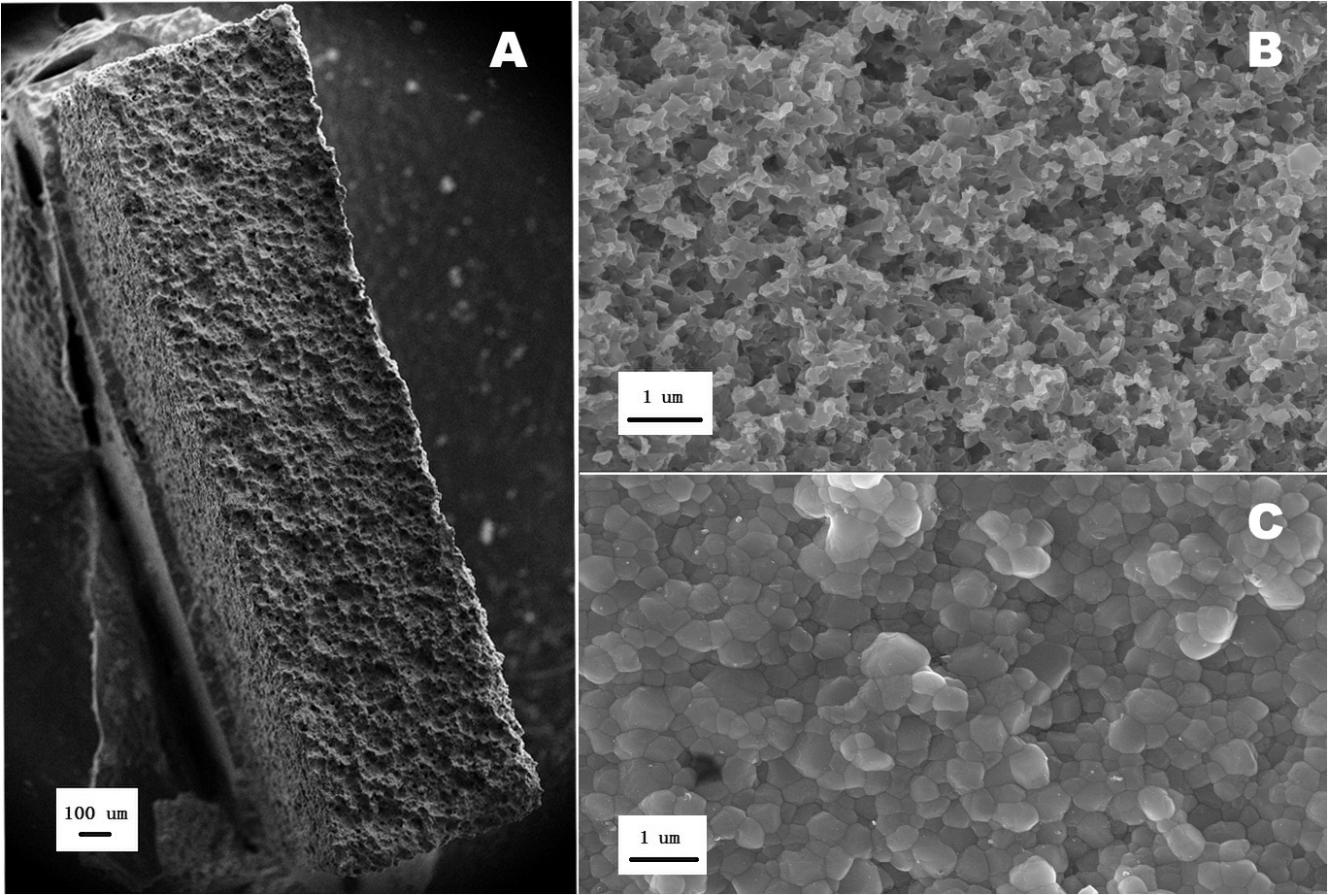
Scanning electron microscopic images of representative specimens from the hydrofluoric (HF) acid-immersed groups (A, B) and the other groups (C). As shown in A and B, a cellular texture with round, shallow concavities can be observed. In contrast, no etched morphology can be observed in C.

SEM observations revealed completely different Y-TZP surface morphologies in the 11 groups. All the HF acid-immersed specimens were evidently etched and exhibited a cellular texture, including the dislodgment of superficial grains, an irregular grain shape, and a decrease in grain size (Fig 2A and 2B, irrespective of the duration of immersion and concentration of HF. Several round, shallow concavities were observed in the 40HF5, 40HF1, and 5HF5 group specimens. However, there were no differences in surface morphology among the control, Cage, AC7, AC14, CI7, and CI14 group specimens (Fig 2C, which exhibited a homogenous fine-grained structure and closed intergrain spaces.

### Characterization of phase transformation

**Fig 3:**
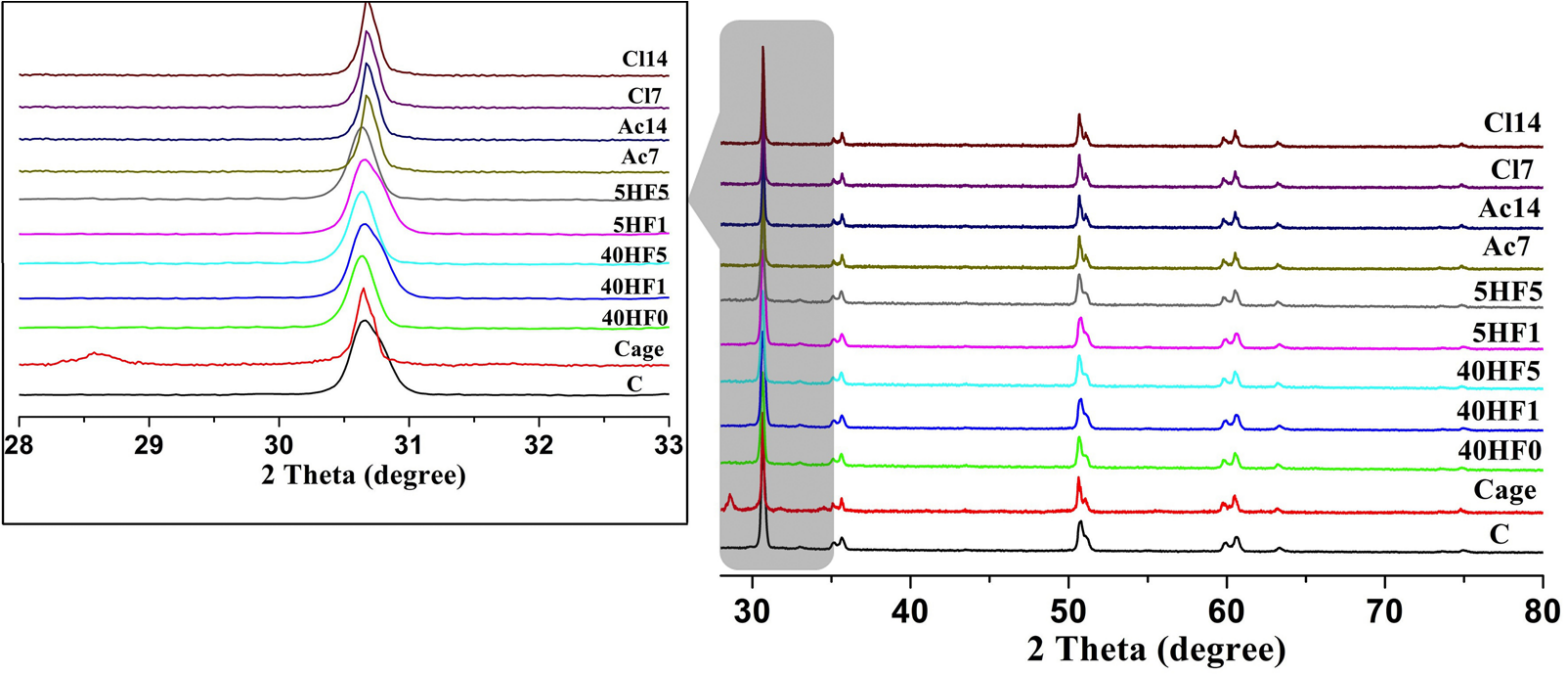
X-ray diffraction (XRD) patterns of specimens from the 11 groups. The m-phase of pure zirconium dioxide is detected at 27.5–28.5° 2θ (arrow) in the low-temperature degradation (LTD) group only. No *m*-ZrO_2_ is detected in the other groups. The left panel shows the XRD patterns at 27–33° 2θ.

As shown in Fig 3, XRD only detected *m*-ZrO_2_ in the Cage group specimens, while no *m*-ZrO_2_ was found in the other groups. The *m*-ZrO_2_ contents could be sequenced according to quantified data from XRD as follows: Cage (19.8 wt%) > 40HF5, 40HF1, 40HF0, 5HF5, 5HF1, AC14, CI14, AC7, CI7, and C (0 wt%).

### Characterization of the sediments after immersion

**Fig 4:**
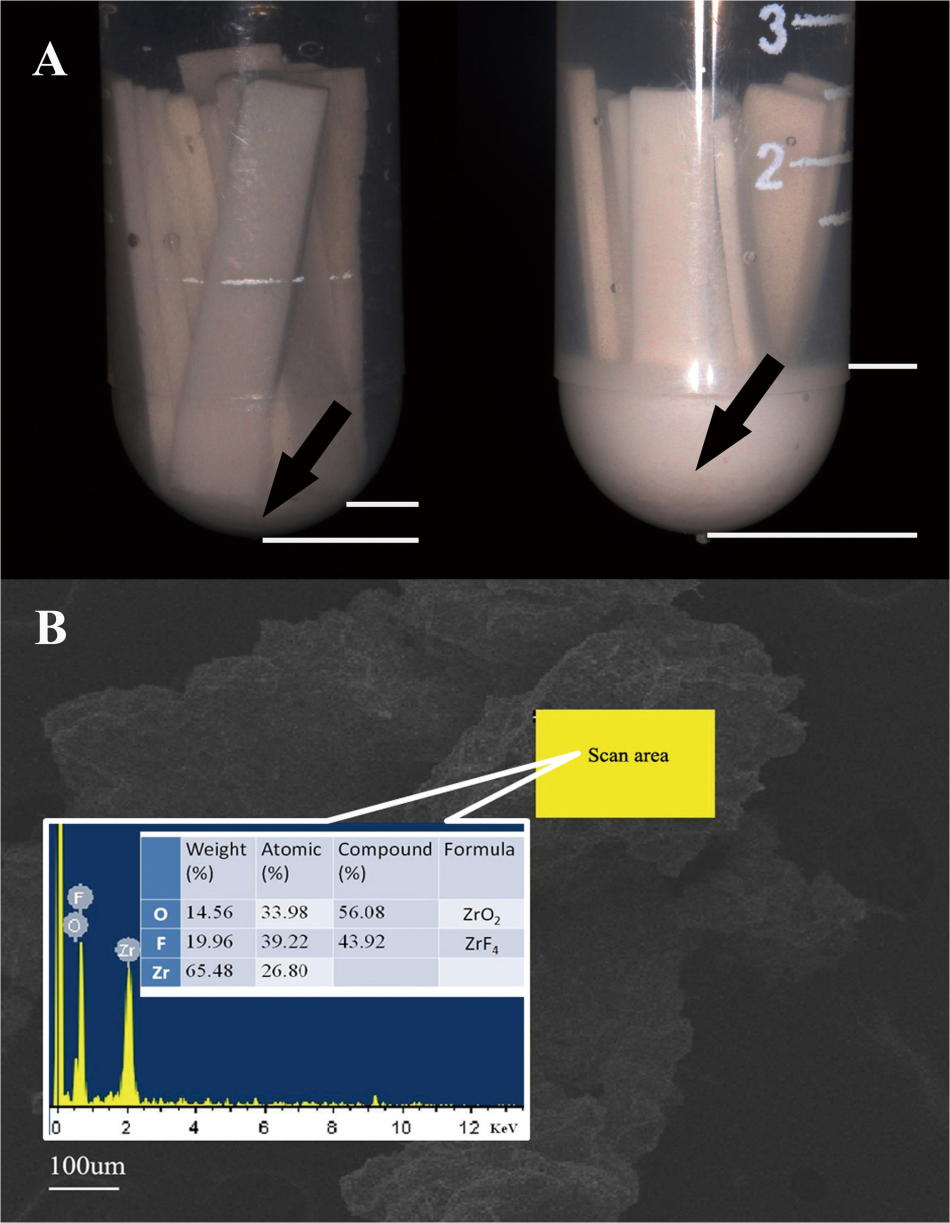
A. Immersion solutions from the 40HF1 (left) and 40HF5 (right) groups. Arrows point to the sediment. The white lines mark the level of the sediment. A large quantity of sediment is observed in the 40HF5 group, while a small visible quantity is observed in the 40HF1 group. 40HF5: immersion in 40% hydrofluoric (HF) acid for 5 days. 40HF1: immersion in 40% HF acid for 1 day. B. Scanning electron microscopy/energy-dispersive X-ray microanalysis (SEM/EDS) findings for sediments formed in the solutions from the HF acid-treated groups.

**Fig 5:**
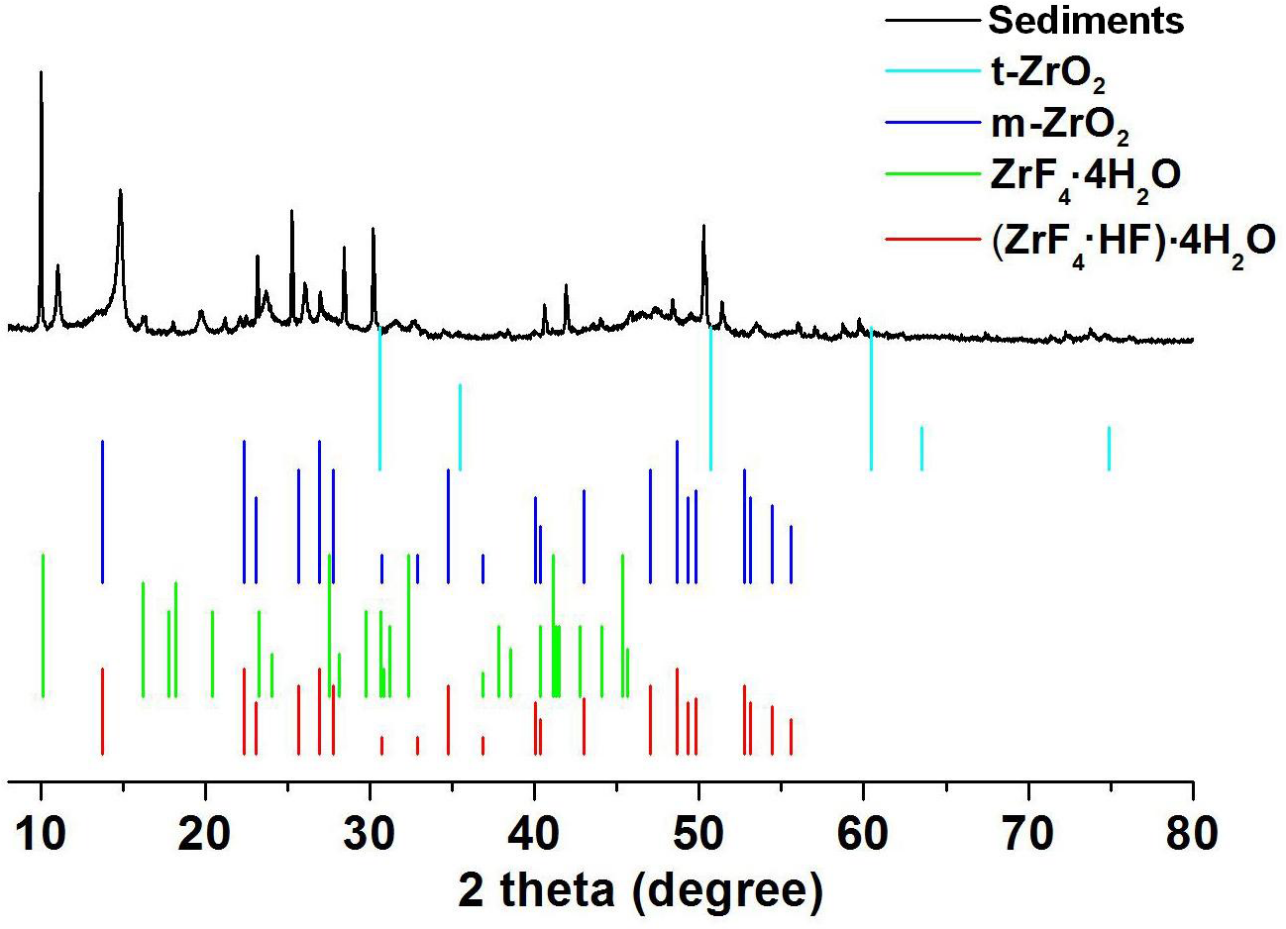
X-ray diffraction (XRD) patterns of the sediments formed in the solutions from the 40% hydrofluoric (HF) acid-immersed groups. The diffraction peaks can be well indexed to *t*-ZrO_2_ (JCPDS 00-001-0750), *m*-ZrO_2_ (JCPDS 00-007-0337), ZrF_4_·4H_2_O (JCPDS 00-019-1486), and (ZrF_4_·HF)·4H_2_O (JCPDS 00-009-0118).

A large quantity of sediment was observed in the immersion solutions from the 40HF5 group, while a small amount of visible sediment was observed in the solutions from the 40HF1 group (Fig 4A). No obvious sediment was observed in the solutions from the 40HF0 and other groups. The composition of the sediments was examined by XRD (Fig 5); the diffraction peaks could be well indexed to *t*-ZrO_2_ (JCPDS 00-001-0750), *m*-ZrO_2_ (JCPDS 00-007-0337), ZrF_4_·4H_2_O (JCPDS 00-019-1486), and (ZrF_4_·HF)·4H_2_O (JCPDS 00-009-0118). According to quantitative analysis of three different scanned regions using SEM/EDS (Fig 4B), the sediments were a mixture of ZrO_2_ (54.08 ± 3.52%) and ZrF_4_ (46.54 ± 3.08%).

## Discussion

Flexural strength, surface hardness, and surface finish are important physical properties that affect the clinical performance of Y-TZP restorations. Flexural strength contributes to fracture resistance, surface hardness to wear resistance, and an excellent surface finish to the prevention of bacterial adhesion and wear of antagonist teeth. Therefore, these parameters were investigated after the treatment of Y-TZP restorations with HF, acetic, and citric acids at ambient temperature in the present study. The Y-TZP specimens immersed in 10% acetic acid and 20% citric acid at ambient temperature did not exhibit any significant changes in flexural strength, surface Vickers hardness, and surface roughness. To determine whether immersion in acetic acid and citric acid would accelerate LTD of Y-TZP, a negative control group that received no treatment and a hydrothermally aged group, which simulated LTD of Y-TZP, were included. Hydrothermal aging at 134°C is frequently employed for accelerated testing of LTD [16]. According to a previous study, 1 hour of in vitro hydrothermal aging at 134°C corresponds to 1 to 2 years of in vivo aging [17]; therefore, the hydrothermal aging conditions adopted in the present study should be equivalent to more than 20 years of in vivo aging. XRD revealed a significant increase of up to 19.8 wt% in *m*-ZrO_2_ contents in the Cage group compared with those in the negative control group. Nevertheless, the present hydrothermal aging condition did not affect the surface Vickers hardness and surface morphology in an adverse manner after 20 h, although it resulted in a slight increase in the flexural strength, which could have been caused by phase transformation toughening. These results are consistent with those reported in previous studies [18–20]. Similarly, in addition to the negligible change in physical properties, the crystalline structure of the Y-TZP specimens immersed in acetic acid and citric acid at ambient temperature showed no change, with XRD detecting no *m*-ZrO_2_ content. These results suggest that immersion in acetic acid and citric acid at ambient temperature does not accelerate *t→m* transformation. However, different conclusions have been reported in previous studies. Egilmez et al. [7] reported that the effects of Y-TZP immersion in 4% acetic acid at 80°C for 168 h resulted in more accelerated aging compared with hydrothermal aging at 134°C and 0.2 MPa for 5 h, which detected 20.89% *m*-ZrO_2_. Ardlin [21] reported that 99% ZrO_2_ was resistant to 168 h of 4% acetic acid reflux without compromising its subsequent flexural strength; nevertheless, increased *m*-ZrO_2_ contents were found [21]. These conflicting results may be a consequence of the different zirconia materials used in the studies. According to some authors, diffusion-controlled transformation of Y-TZP strongly depends on the grain size, i.e., a larger grain size may be a disadvantage during prolonged aging below 100°C in an acidic environment [7,22]. In addition, we also consider different reaction temperatures to be an important reason for the conflicting results. According to Guo et al. [23,24], LTD of Y-TZP is accelerated by the chemical reaction of H_2_O with O^2−^ on the ZrO_2_ surface to form-OH groups, which penetrate the inner part of Y-TZP crystals via grain boundary diffusion to fill the oxygen spaces and form protonic defects. These reactions are easier to execute at higher temperature or higher pressure. Therefore, the lower reaction temperature adopted in the present study did not provide a favorable environment for accelerating LTD of Y-TZP. Our results indicate that acetic acid and citric acid present in drinks and foods do not result in appreciable deterioration of the clinical performance of Y-TZP restorations, although exposure to these acids may not be safe when the temperature is higher than 80°C.

On the other hand, significant changes in one or more of the three parameters, flexural strength, Vickers surface hardness, and surface roughness, were detected in all the HF acid-immersed specimens. Hot acid etching has been attempted to achieve microinterlocking and improve the bond between resin and Y-TZP. This includes etching with HCl/Fe_2_Cl_3_ solution at 100°C, HF acid at 100°C, and nitric acid or sulfuric acid [8]. More recently, Sriamporn et al. found that 9.5% and 48% HF can etch dental zirconia ceramic at 25°C, creating obvious nanoporosities within 2 h and 30 min [10]. Although etching at ambient temperature is encouraged and would be potentially beneficial in the clinical setting, potential damage caused by HF etching of Y-TZP restorations have not been investigated before this study. In the present study, nanoporosities were detected on the surface of all specimens from the five HF acid-treated groups. Accordingly, these five groups showed decreased surface hardness compared with the other groups. The present results also suggest that surface roughness is HF concentration-dependent, because 40% HF immersion resulted in greater surface roughness. This result can be attributed to the higher chemical degradation of Y-TZP in higher concentrations of HF acid, similar to the findings of Sriamporn et al. [10]. These results imply that higher concentrations of HF should be used to etch Y-TZP for lesser chairside time and increased surface roughness. However, the safety of this procedure with regard to restoration quality remains controversial. According to flexural strength testing in the present study, Y-TZP specimens immersed in 5% HF acid for 1 and 5 days did not show any decrease in the flexural strength compared with the control group. On the contrary, the flexural strength of specimens from the 40% HF acid groups decreased, even if the immersion time was only 2 h. Moreover, 5% HF immersion resulted in higher surface Vickers hardness values compared with 40% HF immersion. Together, these results suggest that the adverse effects of HF are aggravated by higher concentrations. Moreover, although only two disintegrated specimens were found, the dimensions of all the other specimens in the 40HF5 and 40HF1 groups were decreased when measured by the digital micrometer, and sediments were found in the immersion solutions from these two groups. In addition, no significant difference in flexural strength and surface hardness values were observed between the 40HF5 and 40HF1 groups. This finding suggests that transformation in the deeper layers of Y-TZP is unlikely to occur during chemical degradation, which is probably limited to the surface layer in direct contact with HF acid. To determine whether HF acid etching can lead to LTD of Y-TZP, we analyzed the sediments that originated from early disintegration of the specimens during immersion in 40% HF for 1 and 5 days and the HF acid-immersed specimens from all five groups. Initially, the authors speculated that immersion in 40% HF acid for 1 or 5 days may accelerate LTD of Y-TZP, causing the dislodgment of superficial grains because of the volume expansion that accompanied *t→m* phase transformation. However, according to XRD analysis, no *m*-ZrO_2_ was detected in all the tested HF-immersed Y-TZP specimens, unlike the findings in the Cage group. However, *m*-ZrO_2_ was detected in the sediments from the 40HF1 and 40HF5 groups. The XRD patterns and SEM/EDS data obtained in the present study also confirmed that the sediments contained ZrO_2_ and ZrF_4_; the latter was probably a reaction product of Y-TZP and HF acid. All these results suggest that chemical degradation caused by immersion in HF acid, and not LTD, plays an important role in the deterioration in flexural strength, surface Vickers hardness, and/or surface roughness. HF acid immersion may not necessarily lead to LTD of Y-TZP. With regard to the presence of *m*-ZrO_2_ in the sediments from the 40HF1 and 40HF5 groups, the reason could be the separation of yttria from Y-TZP within the sediments, with *t→m* transformation occurring because of loss of stabilizer. Nevertheless, the adverse effects caused by 40% HF immersion do not prevent us from drawing the conclusion that 5% HF is safe for etching Y-TZP.

## Conclusions

Within the limitations of the present study, the overall results warrant rejection of the null hypothesis that there are no differences in Y-TZP immersed in HF, acetic, and citric acids for different time periods with respect to *t→m* transformation, destabilization of the crystalline phase, and deterioration of the surface finish and mechanical properties. The following conclusions may be drawn.

1. The flexural strength, surface finish, and surface Vickers hardness of Y-TZP are significantly deteriorated by chemical degradation with 40% HF at ambient temperature.
2. 5% HF can create a roughened surface at ambient temperature without deteriorating the flexural strength of Y-TZP restorations.
3. Acetic acid and citric acid do not deteriorate the flexural strength, surface finish, and surface Vickers hardness of Y-TZP restorations at ambient temperature.
